# Neutrophil differentials in bronchoalveolar lavage fluid in bottlenose dolphins (*Tursiops truncatus*) and beluga whales (*Delphinapterus leucas*) during treatment of respiratory infection: a preliminary study

**DOI:** 10.1007/s11259-026-11112-8

**Published:** 2026-02-20

**Authors:** Takashi Kamio, Yukako Odani, Koji Kanda, Tomoko Mori, Yuichiro Akune, Wataru Ohtomo, Masanori Kurita, Ayaka Okada, Yasuo Inoshima

**Affiliations:** 1 Port of Nagoya Public Aquarium, Nagoya Port Foundation, 1-3 Minato-machi, Minato-ku, Nagoya, 455-0033 Aichi Japan; 2 Laboratory of Food and Environmental Hygiene, Joint Department of Veterinary Medicine, Gifu University, 1-1 Yanagido, Gifu, 501-1193 Japan; 3 Joint Graduate School of Veterinary Sciences, Gifu University, 1-1 Yanagido, Gifu, 501-1193 Japan

**Keywords:** Beluga whale, Bottlenose dolphin, Bronchoalveolar lavage fluid, Respiratory infections

## Abstract

Respiratory infections are major health concerns in wild and managed cetaceans, yet reliable methods for assessing lower respiratory tract inflammation are limited. We aimed to evaluate the clinical utility of bronchoalveolar lavage fluid (BALF) neutrophil differentials in three managed bottlenose dolphins (*Tursiops truncatus*) and three managed beluga whales (*Delphinapterus leucas*) undergoing treatment for respiratory infections. Infection status was monitored by assessing isolated bacteria and fungi from BALF, BALF neutrophil differentials, and inflammatory markers in the blood, such as total leukocyte counts, plasma fibrinogen levels, and serum iron levels. In animals with respiratory bacterial and/or fungal infections alone, both BALF neutrophil differentials (47.3–55.7%) and blood inflammatory markers were elevated. Following antimicrobial treatment, the differentials decreased (0.8–3.8%), and blood inflammatory markers also returned to normal ranges. Contrastingly, in animals with concurrent conditions, such as respiratory bacterial and/or fungal infections accompanied by wounds on the tail fin or infection-induced iron deficiency anemia, persistent blood inflammatory markers were observed after antimicrobial treatment. Despite this, BALF neutrophil differentials, which are specific to respiratory inflammation, decreased in response to treatment (3.1–21.6%). Moreover, microorganisms isolated from blow samples from these animals were not consistent with those isolated from BALF at the same time. These findings suggest that BALF reflects localized respiratory infection more accurately than the inflammatory markers in the blood, and BALF examination is useful for diagnosing microbial infections in the lower respiratory tract in cetaceans. This approach may improve clinical decision-making and therapeutic outcomes in managed cetacean populations worldwide.

**Supplementary information:**

The online version contains supplementary material available at 10.1007/s11259-026-11112-8.

## Background

Respiratory infections pose life-threatening risks to both wild and managed cetaceans, including bottlenose dolphins (*Tursiops truncatus*) (Nollens et al. 2018; Reidarson et al. 2018; Tryland et al. 2018; Van Bonn and Dover 2018) and beluga whales (*Delphinapterus leucas*) (Nollens et al. 2018; Reidarson et al. 2018; Tryland et al. 2018; Van Bonn and Dover 2018). Pathogenic microorganisms, such as *Staphylococcus aureus*, which frequently cause pneumonia in cetaceans (Tryland et al. 2018; Venn-Watson et al. 2012), can easily invade the lower respiratory tract due to the overall respiratory design of cetaceans, which enables the rapid exchange of large volumes of air (Venn-Watson et al. 2012).

Respiratory infections in cetaceans can be diagnosed using thoracic radiography and/or computed tomography (CT) (Dennison and Saviano 2018), ultrasonographic imaging (Martony et al. 2017; Smith et al. 2012), and pulmonary function testing (Borque-Espinosa et al. 2020); however, these diagnostic modalities cannot detect pathogenic microorganisms. Detection of pathogenic microorganisms is possible by blow samples, typically collected via swabs or chuff samples (Ohno et al. 2019); however, they are considered unreliable for detecting pathogenic microorganisms in the lower respiratory tract (Kamio et al. 2025b; Nollens et al. 2018; Venn-Watson et al. 2012). Bronchoscopy has been used to assess respiratory conditions in cetaceans for several decades (Kamio et al. 2025b; Nollens et al. 2018; Tsang et al. 2002; Van Bonn and Dover 2018). This technique contributed to monitoring the course of invasive respiratory aspergillosis, leading to successful treatment in a bottlenose dolphin (Bunskoek et al. 2017). In humans (Meyer 2007) and terrestrial animals (Davis and Sheats 2019; Jolie et al. 2000), bronchoalveolar lavage fluid (BALF) obtained through bronchoscopy is a crucial tool for diagnosing lower respiratory infections by analyzing leukocyte differentials. However, in cetaceans, there are few controlled studies or established standard ranges for leukocyte differential (Hawkins et al. 1997; Van Bonn and Dover 2018), because bronchoscopy and BALF require specialized techniques and equipment, procedures are technically difficult once the scope passes the blowhole, and out-of-water restraint or sedation may pose risks (Van Bonn and Dover 2018).

Hawkins et al. (1997) collected BALF twice from a stranded Atlantic bottlenose dolphin without respiratory infection and evaluated leukocyte differentials. Based on the findings, they concluded that if mononuclear cells account for approximately 70% of leukocytes in BALF, respiratory infection is unlikely. In that case, the BALF neutrophil differential was 9% and 26%, respectively (Hawkins et al. 1997).

Inflammatory markers in the blood are commonly used to diagnose respiratory infections in bottlenose dolphins and beluga whales (Nollens et al. 2018). These markers include an increased total leukocyte count (standard ranges: 5000–9000/µL in bottlenose dolphins, 5000–9500/µL in beluga whales), elevated plasma fibrinogen level (standard ranges: 170–280 mg/dL in bottlenose dolphins, < 70 mg/dL in beluga whales), and decreased serum iron levels (standard ranges: 120–340 µg/dL in bottlenose dolphins, 195–380 µg/dL in beluga whales) (Online Resource S1). Serum iron level is a useful marker for infections, as it may normalize early during successful treatment (Nollens et al. 2018). While these markers are not specific to respiratory conditions, chronic low-grade pneumonia is relatively common in cetaceans and often detectable only through slight elevations in these parameters or mild non-regenerative anemia; therefore, pneumonia should be considered as a working diagnosis in cases of illness of unknown origin (Nollens et al. 2018).

At the Port of Nagoya Public Aquarium, respiratory infection was suspected when animals exhibited anorexia and/or fever (defined as ≥ 0.5 °C above the animal’s average rectal temperature), along with at least two of the following blood criteria: for bottlenose dolphins, leukocyte count > 9,000/µL, fibrinogen > 280 mg/dL, or serum iron < 120 µg/dL; for beluga whales, leukocyte count > 9,500/µL, fibrinogen > 70 mg/dL, or serum iron < 195 µg/dL. In such cases, bronchoscopy was performed (Online Resource S1). If treatment for respiratory infection is based solely on microorganisms detected in BALF, contamination may result in unwarranted therapy or an extended treatment course. To prevent such situations, it has become urgent to determine whether the BALF neutrophil differential is useful as an indicator for initiating and terminating treatment.

In this study, we aimed to clarify the utility of neutrophil differential analysis in BALF for diagnosing lower respiratory infections in managed bottlenose dolphins and beluga whales, as well as to evaluate how inflammatory markers in the blood change during the course of treatment for these infections.

## Case presentation

We performed bronchoscopies and collected BALF (Kamio et al. 2025b) from three bottlenose dolphins (ID: TT-HP, TT-LL, and TT-SR) and three beluga whales (ID: DL-9, DL-11, and DL-12) under light sedation as described previously (Kamio et al. 2025b) at the Port of Nagoya Public Aquarium between December 2022 and April 2025. Briefly, sedation was administered using a combination of intramuscular midazolam (Sandoz, Tokyo, Japan) and intramuscular butorphanol (Meiji Seika Pharma Co., Ltd., Tokyo, Japan) 30 min before bronchoscopy (Kamio et al. 2025b), and/or oral diazepam (Tsuruhara, Osaka, Japan) with or without oral tramadol (Pfizer, Tokyo, Japan) administered 120 min before the procedure. The doses administered to each animal are provided in Online Resource S2. Blow samples were collected to compare the isolated microorganisms with those obtained from BALF (Kamio et al. 2025b). The condition of the cetaceans is described in Online Resource S3. Bronchoscopy was performed using an endoscope with an outer diameter of 9.3 mm (VQ-9143 C, working length 1,400 mm; Olympus, Tokyo, Japan) for bottlenose dolphins, and an endoscope with the same outer diameter (VQ-9143 C, working length 3,000 mm; Olympus) for beluga whales. During bronchoscopy, air was continuously insufflated through the endoscope until it passed the epiglottis to avoid upper respiratory tract contamination. If contamination was suspected, the endoscope was removed, disinfected, and the procedure was resumed. Approximately 50 mL of sterilized saline was infused into each impassable point of the left and right principal bronchi and the tracheal bronchus (Kamio et al. 2025b) via the endoscope water supply channel, and more than 30% of the infused saline was retrieved through the suction channel connected to the endoscope suction pump. BALF samples from the three impassable points were pooled for analysis. BALF was collected in a 50-mL tube and centrifuged at 2,600 × g for 15 min. The obtained precipitates were used for microbial culture and cytological examination. Cytological preparations were made within 2 h after sampling to minimize cellular degradation. Because absolute cell counts were not calculated, cellular differentials were expressed as percentages to minimize the influence of variations in recovered volume. For bacterial culture, the precipitates were inoculated onto a Seed Swab (Eiken Chemical Co., Ltd., Tokyo, Japan), and for fungal culture, the precipitates were streaked onto Sabouraud dextrose agar plates (BD Japan, Tokyo, Japan).

For blow samples, specimens were collected non-invasively (*n* = 5/sampling). For bacterial culture, blow samples were collected directly onto sterilized plates and inoculated in the same manner as BALF onto the Seed Swab. For fungal culture, blow samples were collected directly onto Sabouraud dextrose agar plates and inoculated as described above. The animal experimental procedures were reviewed and approved by the Gifu University Animal Care and Use Committee (approval numbers: 2020 − 264 and AG-P-C-20240001).

For bacteria, clinical testing laboratories performed bacterial identification and antimicrobial susceptibility testing. Antimicrobial susceptibility testing was performed using the broth microdilution assay and disk diffusion methods, and the results were interpreted according to the Clinical and Laboratory Standards Institute (CLSI) M100 guidelines. For fungi, the plates were incubated in an IC-450 A incubator (AS ONE Corp., Osaka, Japan) at 37 °C for 48 h and sent to the clinical testing laboratories to perform *Candida* spp. (broth microdilution assay, CLSI M27) and filamentous fungi (broth microdilution assay, CLSI M38) identification and antifungal susceptibility testing, respectively. Antimicrobial treatments for respiratory infections were selected based on these susceptibility findings. Blow samples were collected to assess potential upper respiratory contamination (Kamio et al. 2025b).

We prepared BALF samples by smearing them on slides, fixing with methanol, and staining with Hemacolor (Merck, Darmstadt, Germany). For each slide, 100–150 leukocytes were counted to determine differentials (Meyer 2007). Absolute neutrophil counts in BALF vary depending on lavage volume, retrieval efficiency (30–60%), and slide loading. Although we attempted to standardize these conditions, the remaining variability was too great to allow reliable comparisons. No reference values have been established for BALF neutrophil differentials in cetaceans. According to Hawkins et al. (1997), we provisionally defined a BALF neutrophil differential of < 30% as the reference threshold in the present study.

Inflammatory markers in the blood were measured for all six animals (Kamio et al. 2025a). Serum iron was measured using Nitroso PSAP by the Nagoya Clinical Center. Plasma voriconazole concentrations (target trough concentration: 3.0 µg/mL (Kamio et al. 2025a) were measured using liquid chromatography–tandem mass spectrometry by SRL, Inc. (Tokyo, Japan). We determined leukocyte differentials by counting 150–200 cells on May-Grünwald-stained smears (Sigma-Aldrich, Tokyo, Japan). Neutrophil, eosinophil, lymphocyte, and monocyte counts (/µL) were then calculated from total leukocyte counts and differential percentages.

## Animals with respiratory infection and other health disorders (TT-HP and DL-11)

### TT-HP

**Fig. 1:**
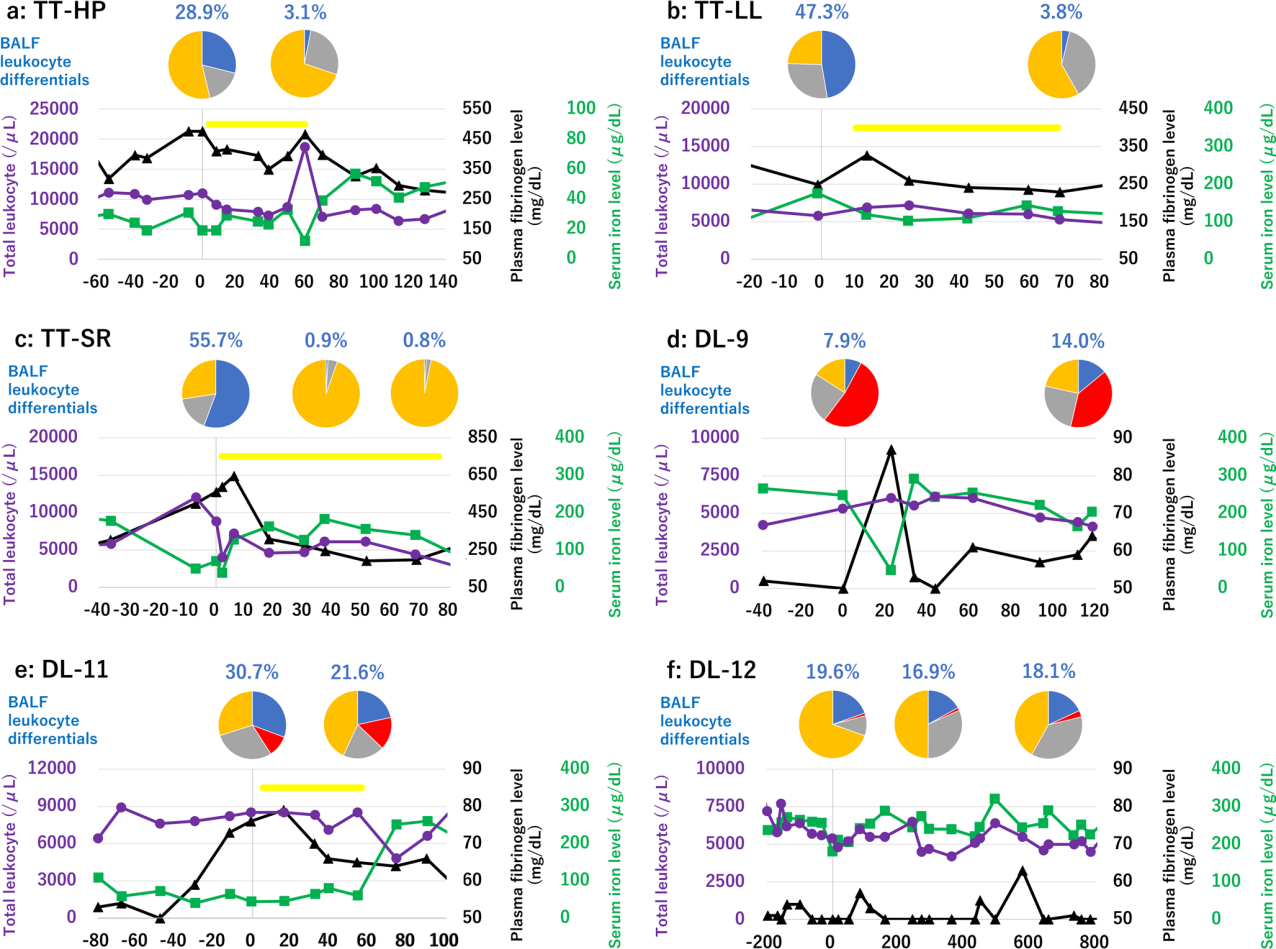
Respiratory infection treatments in three bottlenose dolphins (*Tursiops truncatus*) (a TT-HP, b TT-LL, and c TT-SR) and three beluga whales (*Delphinapterus leucas*) (d DL-9, e DL-11, and f DL-12) Upper panels displayed bronchoalveolar lavage fluid (BALF) leukocyte differentials. Neutrophil (blue, percentage is shown); eosinophil (red); lymphocyte (grey); and macrophage (orange). Lower panels showed total leukocyte (/µL, purple circle), plasma fibrinogen level (mg/dL, black triangle), and serum iron level (µg/dL, green square). Yellow bars indicated the duration of respiratory infection treatment (1a: Day 4–59, 1b: Day 10–68, 1c: Day 2–76, 1e: Day 5–56).TT-HP and TT-SR had respiratory infections; TT-HP and DL-11 had respiratory infections accompanied by iron-deficiency anemia, with TT-HP or without DL-11 tail fin wound due to scratching as described in the text, respectively; and DL-9 and DL-12 were clinically healthy In TT-HP, TT-LL, TT-SR, and DL-11, respiratory infections were suspected based on the results of the clinical examinations as described in the text, and the bronchoscopy was therefore performed. The first day on which a respiratory infection was diagnosed by bronchoscopy in each animal was designated as Day 0, and dates prior to this were indicated with negative numbers. For animals that underwent bronchoscopy as part of routine health monitoring (DL-9 and DL-12), the first bronchoscopy performed during the study period was designated as Day 0

 TT-HP, a bottlenose dolphin, had congenital scoliosis resulting in unstable swimming, which consequently caused recurrent tail fin wounds due to scratching, presented with leukocytosis (9100–11100/µL) and iron deficiency anemia (serum iron level, 19–31 µg/dL; hematocrit, 34.6–38.4%) from Day − 81–0. On Day 0, inflammatory markers in the blood (total leukocyte count, 11000/µL; plasma fibrinogen level, 476 mg/dL; hematocrit, 37.6%; serum iron level, 19 µg/dL) with fever (37.3℃) were observed, and bronchoscopy was prescribed. *Escherichia coli*, *Proteus hauseri*, and *Candida albicans* were isolated from the BALF (Online Resource S4). Although the BALF neutrophil differential (28.9%) was below the reference threshold of 30%, the presence of phagocytosed bacteria and fungi confirmed a diagnosis of respiratory infection (Fig. 1a; Table 1). Minocycline (Sawai, Osaka, Japan) and voriconazole (DSEP, Tokyo, Japan) were initiated based on the findings of antibacterial susceptibility testing (Online Resource S4 and S5). While blood inflammatory markers remained (total leukocyte count, 18700/µL; plasma fibrinogen level, 467 mg/dL; hematocrit, 43.5%; serum iron level, 12 µg/dL), the antimicrobial therapy led to normalization of BALF neutrophil differentials (3.1%) on Day 59 (Fig. 1a; Table 1). While *E. coli* and *Proteus hauseri* were continuously isolated from BALF, antimicrobial therapy for respiratory infection was discontinued. Recurrent wound-related inflammation was treated with polyhexamethylene biguanide with betaine solution (Kamio et al. 2025a) and oral minocycline, resulting in the wound’s complete healing on Day 141. Inflammatory markers in the blood (total leukocyte count, 7900/µL; plasma fibrinogen level, 291 mg/dL; hematocrit, 52.0%; serum iron level, 173 µg/dL) were normalized on Day 190.

**Table 1:** Sample results of Bronchoalveolar lavage fluid and inflammatory markers in the blood

ID	Examined day (Day)	Bronchoalveolar lavage fluid				Hematological examination					Plasma fibrinogen (mg/dL)	Hematocrit (%)	Serum iron (μg/dL)
		Leukocyte differentials (%)				Leukocyte counts (/μL)							
		Ne	Eo	Ly	Mφ	Total	Ne	Eo	Ly	Mo			
Bottlenose dolphins													
TT-Hp	-525	4.2	0.0	19.5	76.3	5600	3897.6	700.0	968.8	33.6	350	40.5	30
	0	28.9	0.0	17.4	53.7	11,000	9295.0	825.0	825.0	55.0	476	37.6	19
	59	3.1	0.0	27.1	69.8	18,700	15,801.5	897.6	1608.2	392.7	467	43.5	12
	141	NA	NA	NA	NA	8,100	4868.1	1449.9	1636.2	145.8	273	47.5	51
	190	NA	NA	NA	NA	7,900	4937.5	1303.5	1572.1	86.9	291	52.0	173
TT-LL	-735	0.0	0.0	3.9	96.1	7800	6481.8	405.6	842.4	70.2	309	47.9	189
	-20	48.9	0.0	19.3	32.8	6600	5885.5	758.4	1137.6	118.5	302	53.7	108
	0	47.3	0.0	28.1	24.6	5800	6098.8	308.1	1406.2	86.9	249	53.2	176
	70	3.8	0.0	38.0	58.2	5300	6004.0	766.3	1003.3	47.4	229	59.7	128
TT-Sr	0	55.7	0.0	16.8	27.5	8800	6256.8	765.6	1575.2	202.4	560	42.9	70
	2	NA	NA	NA	NA	4000	1728.0	808.0	1268.0	196.0	588	42.6	39
	37	0.9	0.0	4.5	94.6	6100	2720.6	1427.4	1915.4	36.6	245	50.7	183
	72	0.8	0.0	2.4	96.9	4400	2002.0	686.4	1658.8	52.8	198	48.9	140
Beluga whales													
DL-9	0	7.9	52.4	23.8	15.9	5300	4250.2	774.2	2788.7	86.9	<50	58.6	248
	112	14.0	39.7	24.8	21.5	4400	3246.9	655.7	3855.2	142.2	59	55.8	165
DL-11	-267	20.9	16.2	25.7	37.2	7900	5135.0	695.2	1951.3	118.5	52	53.6	120
	0	30.7	10.2	29.2	29.9	8500	4862.0	1283.5	2099.5	255.0	76	46.0	44
	56	21.6	15.5	19.8	43.1	8500	4156.5	1224.0	2941.0	178.5	65	50.3	60
	91	NA	NA	NA	NA	6600	4276.8	402.6	1722.6	198.0	66	52.4	260
DL-12	0	19.6	1.3	9.5	69.6	5400	2953.8	226.8	2089.8	129.6	<50	60.1	181
	84	17.8	5.6	30.8	45.8	6000	3096.0	534.0	2334.0	36.0	57	55.9	243
	294	16.9	1.4	31.8	49.9	4700	2561.5	79.9	1950.5	108.1	<50	58.6	241
	433	23.7	2.5	33.1	40.7	5100	2830.5	127.5	2111.4	30.6	<50	60.6	221
	658	18.1	3.1	37.0	41.8	5000	3626.1	252.8	3768.3	252.8	<50	56.2	290

In TT-HP, TT-LL, TT-SR, and DL-11, respiratory infections were suspected based on their results of the clinical examinations as described in the text, the bronchoscopy was carried out. The first day on which respiratory infection was diagnosed by bronchoscopy in each animal was designated as Day 0, and dates prior to this were indicated with negative numbers. For animals that underwent bronchoscopy as part of routine health monitoring (DL-9, DL-12), the first bronchoscopy performed during the study period was designated as Day 0. Ne, neutrophil; Eo, eosinophil; Ly, lymphocyte; Mφ, macrophage; Mo, monocyte, and NA, not applicable

## DL-11

DL-11, a beluga whale with recurrent iron deficiency anemia (hematocrit, 43.9–51.2%; serum iron level, 40–108 µg/dL), was unresponsive to oral ferrous sulfate (Day − 109–Day − 68). A bronchoscopy was performed to investigate the persistent hypoferremia (hematocrit, 46.0%; serum iron level, 44 µg/dL) on Day 0, because respiratory infection had a possibility to be the cause of the low serum iron level. Because priority was given to identifying the pathogenic microorganisms, examinations, such as lung radiography and ultrasonographic imaging, were not performed. Combined with a high plasma fibrinogen level (76 mg/dL), a respiratory infection was confirmed by high BALF neutrophil differential (30.7%) (Fig. 1e; Table 1) and the isolated *Pseudomonas aeruginosa*, *E. coli*, *Morganella morganii*, *Shewanella putrefaciens*, and *Vibrio alginolyticus* from BALF (Online Resource S4). Based on the findings of antibacterial susceptibility testing (Online Resource S5), antibiotics were switched from amoxicillin (Fujita, Tokyo, Japan) with potassium clavulanate (GSK, Tokyo, Japan) to levofloxacin (Sawai, Osaka, Japan) on Day 5. Amikacin (FujiPharma, Toyama, Japan) nebulization therapy was added after the completion of voluntary behavior training for DL-11. The antibacterial therapy controlled the infection on Day 56, with normalized BALF neutrophil differentials (21.6%), hematocrit (50.3%), and plasma fibrinogen level (65 mg/dL), although *E. coli* was still isolated from BALF. After the respiratory bacterial infection was concluded on Day 56, the serum iron level remained low (60 µg/dL). Subsequent oral ferrous sulfate therapy normalized hematocrit (52.4%) and serum iron level (260 µg/dL) on Day 91.

## Animals with respiratory infection (TT-LL and TT-SR)

### TT-LL

TT-LL was suspected of having respiratory infections based on fever (37.0℃) and elevated inflammatory markers in the blood (plasma fibrinogen level, 302 mg/dL; serum iron level, 108 µg/dL). Elevated BALF neutrophil differentials (48.9%) were observed, but no microorganisms were isolated on Day − 20 (Fig. 1b, and Table 1). *C. albicans* was isolated from BALF on Day 0 (BALF neutrophil differentials, 47.3%) (Online Resource S4). Day 0 was defined as the day when each animal was diagnosed with a respiratory infection. Voriconazole was initiated based on the findings of antifungal susceptibility testing (Online Resource S5). Over the course of treatment, plasma fibrinogen level (229 mg/dL), serum iron level (128 µg/dL), and BALF neutrophil differential (3.8%) returned to their normal ranges; no microorganisms were isolated from the BALF on Day 70. The infections were considered controlled, and medications were discontinued on Day 68 (plasma voriconazole concentrations remained until Day 84).

### TT-SR

TT-SR was suspected of having respiratory infections based on anorexia (57.9% of planned diet per day, from Day − 8), fever (37.0℃), and elevated inflammatory markers in the blood (plasma fibrinogen level, 560 mg/dL; serum iron level, 70 µg/dL). Minocycline was administered prior to bronchoscopy but was discontinued due to the development of leukopenia (total leukocyte count, 4000/µL). Elevated BALF neutrophil differentials (55.7%) were observed (Fig. 1c; Table 1). *E. coli* was isolated from BALF on Day 0 (Online Resource S4). Faropenem (Maruho, Osaka, Japan) and Levofloxacin were initiated based on the findings of antibacterial susceptibility testing (Online Resource S4 and S5). Over the course of treatment, plasma fibrinogen level (245 mg/dL), serum iron level (176 µg/dL), and BALF neutrophil differential (0.9%) returned to their normal ranges, but *E. coli* was isolated from the BALF on Day 37, and antibiotics were continued. On Day 72, the infections were considered controlled, and medications were discontinued because no microorganisms were isolated from BALF, and the plasma fibrinogen level (198 mg/dL), serum iron level (140 µg/dL), and BALF neutrophil differential (0.8%) remained within the normal ranges established for the present study.

## Animals without respiratory infection (TT-HP, Day − 525; TT-LL, Day − 735; DL-9; DL-11, Day − 267; and DL-12)

Due to the limited number of animals, data from individuals that were not affected when bronchoscopy was performed, but subsequently developed respiratory infections more than 180 days later, were also referenced for comparison.

TT-HP (Day − 525) had an elevated plasma fibrinogen level (350 mg/dL) and decreased serum iron level (30 µg/dL), suspected to be secondary to dermatitis. TT-LL (Day − 735) developed an elevated plasma fibrinogen level (309 mg/dL), secondary to fungal dermatitis of the tail fluke caused by dematiaceous fungi and *Fusarium oxysporum* (Kamio et al. 2025a). Respiratory infection was excluded because the BALF neutrophil differentials remained low (TT-HP, 4.2%; TT-LL, 0.0%) (Table 1).

Two beluga whales (DL-9 and DL-12) were clinically healthy and showed normal levels of inflammatory markers in the blood. Bronchoscopies were performed as part of routine physical examinations, and BALF samples were collected. The BALF neutrophil differentials consistently remained ˂30% (DL-9, 7.9–14.0%; DL-12, 16.9–23.7%) (Fig. 1d, f; Table 1) and no microorganisms were isolated from BALF (Online Resource S4).

A beluga whale (DL-11, Day − 267) presented with a depressed serum iron level (120 µg/dL), while the other inflammatory markers remained normal (total leukocyte count, 7900/µL; plasma fibrinogen level, 52 mg/dL; hematocrit, 53.6%; serum iron level, 120 µg/dL). The BALF neutrophil differential (20.9%) showed similar levels to those of DL-9 and − 12 (Table 1).

## Discussion and conclusions

This study highlights the diagnostic and monitoring value of combining BALF analysis with blood inflammatory markers in cetaceans. Because identification of the pathogenic microorganisms was prioritized, imaging examinations were not performed in the present case; however, combining these approaches with lesion localization using thoracic radiography (Dennison and Saviano 2018) and/or ultrasonographic imaging (Martony et al. 2017; Smith et al. 2012) may facilitate more precise management, including the monitoring of treatment efficacy. Although both bacterial (*E. coli* and *P. aeruginosa*) and fungal *(C. albicans*) pathogens were detected in BALF, no clear differences in the BALF neutrophil response were noted between the two pathogen types. Obvious clinical signs, such as cough, discharge, and abnormal sounds from the respiratory tract, were not observed.

Bronchoscopy and subsequent BALF analysis allow for accurate evaluation of lower respiratory infections, as observed in humans (Meyer 2007) and terrestrial animals, such as pigs (Jolie et al. 2000) and horses (Davis and Sheats 2019). At the cellular level, recruitment of neutrophils to the lung plays a key role in responding to infection. In the lung, endothelial cells upregulate P-selectin to bind circulating naive neutrophils, and β2 integrin on neutrophils subsequently binds ICAM-1 on the endothelium, allowing extravasation (Giacalone et al. 2020).

In dolphins with respiratory infections (TT-LL and TT-SR), both BALF neutrophil differentials and blood inflammatory markers worsened, with the inflammatory markers in the blood normalizing in parallel with an improvement (i.e., reduction) in BALF neutrophil differentials as the microorganisms disappeared. At the time of bronchoscopy, dolphins presenting with dermatitis only (TT-HP, Day − 525; TT-LL, Day − 735) showed inflammatory responses in the blood, whereas BALF neutrophil differentials remained stable. These individuals were initially free of respiratory disease, but developed respiratory infections more than 180 days later. In bottlenose dolphins with both respiratory infection and dermatitis (TT-HP), BALF neutrophil differentials increased, indicating lower respiratory inflammation, and then the neutrophil differentials decreased with antimicrobial treatment. However, inflammatory markers in the blood remained elevated due to systemic conditions. These findings emphasize the lower respiratory tract specificity of BALF. Similar patterns were observed in beluga whales, indicating that combining BALF and blood markers is effective not only in bottlenose dolphins but also in other cetaceans. Notably, BALF neutrophil differentials occasionally remained below 30%, similar to values observed in animals without respiratory infection, despite the isolation of microorganisms, suggesting that microbial presence does not always elicit a detectable local inflammatory response. Consequently, antimicrobial therapy was discontinued when BALF neutrophils remained below 30% after treatment, although bacterial pathogens (*E. coli* and *P. aeruginosa* in TT-HP and *E. coli* in DL-11) were detected from BALF. These pathogens were considered contaminants originating from the upper respiratory tract and/or the environment. The decreased neutrophil differentials (< 30%) in BALF correlated with the resolution of active infection.

Comparisons with human studies further support these findings. In pulmonary *Mycobacterium avium* complex infection, high BALF neutrophil differentials are associated with greater radiological severity and disease progression (Inomata et al. 2018). In human sarcoidosis, BALF neutrophil differentials, rather than blood neutrophils, better reflect pulmonary abnormalities (Feng et al. 2022). These results are consistent with our observations that BALF more specifically represents local respiratory inflammation, while blood markers can be influenced by systemic or extra-respiratory conditions.

Isolated microorganisms from blow sample cultures were not completely consistent with those from BALF, often showing a higher number of bacterial species (Online Resource S4), which may be explained by factors, such as upper respiratory tract contamination, seawater dilution, or transient environmental microorganisms. In addition, bacteria and fungi detected in blow samples were also present in healthy animals and in cases where treatment for lower respiratory tract disease had been completed, with both BALF neutrophil differentials and blood inflammatory markers having returned to reference ranges, suggesting that these microorganisms were not directly responsible for inflammation. Therefore, while blow sampling is useful as a non-invasive screening tool, BALF remains a more reliable diagnostic specimen for identifying lower respiratory tract pathogens.

Finally, while reference values for BALF neutrophil differentials in cetaceans remain limited, our study suggests that values exceeding 30% may indicate lower respiratory infections in both bottlenose dolphins and beluga whales, providing a practical criterion for future clinical applications. Importantly, in the present study, one bottlenose dolphin exhibited a BALF neutrophil differential of 28%, yet respiratory infection was confirmed based on the presence of phagocytosed bacteria and fungi. This finding suggests that values slightly below 30% may still warrant clinical consideration, particularly when cytological evidence of infection is present. Notably, this threshold is somewhat consistent with findings in humans, where a BALF neutrophil differential ≥ 16% was reported as optimal for distinguishing infectious from non-infectious diseases (Li et al. 2024). Moreover, studies on pigs have demonstrated an increase in both BALF neutrophil differential and the number of neutrophils in complete blood count (CBC) in unhealthy pigs compared to healthy pigs (Jolie et al. 2000). In horses, BALF neutrophil differentials of 7–19% and ≥ 20% were categorized as mild-to-moderate and severe neutrophilic inflammation, respectively (Davis and Sheats 2019). However, this study was limited by the small sample size (*n* = 6), which reflects the inherent challenges of conducting clinical research in managed cetaceans. Further studies with larger sample sizes are required to validate and refine these diagnostic thresholds. Establishing standardized BALF thresholds with larger cohorts will enhance diagnostic accuracy and treatment monitoring in cetaceans.

In the present study, we demonstrated that combining BALF analysis with inflammatory markers in the blood improves the diagnosis and monitoring of respiratory infections in cetaceans, especially in the lower respiratory tract. In uncomplicated respiratory infections, both BALF and inflammatory markers in the blood improved concurrently. However, concurrent systemic problems caused discordance. Moreover, microorganisms isolated from BALF may not always indicate true inflammation due to the possibility of contamination, and blow sample cultures were useful to assess BALF’s contamination from the upper respiratory tract, and less reliable for detecting pathogens in the lower respiratory tract. These findings highlight the value of BALF and inflammatory markers in the blood for accurate diagnosis and treatment monitoring.

## Supplementary Information

Below is the link to the electronic supplementary material.

Supplementary Material 1

## Data Availability

No datasets were generated or analyzed during the current study.
