# Supplementary material for: Neutrophil differentials in bronchoalveolar lavage fluid in bottlenose dolphins (Tursiops truncatus) and beluga whales (Delphinapterus leucas) during treatment of respiratory infection: a preliminary study
Source: Vet Res Commun. 2026 Feb 20;50(2):163. doi: 10.1007/s11259-026-11112-8 (PMC12923404; doi:10.1007/s11259-026-11112-8)
Supplement: Supplementary file 1 — Supplementary Material 1 [file 11259_2026_11112_MOESM1_ESM.pdf]

Online Resource S1. Standard ranges in the blood in bottlenose dolphins (*Tursiops truncatus*) and beluga whales (*Delphinapterus leucas*)

Contents	Unit	Bottlenose dolphins		Beluga whales	
		Bossart et al., 2001	PNPA [1)]	Bossart et al., 2001	PNPA
Total leukocyte in the blood	/µL	5000–9000	NA [2)]	5000–9500	NA
Plasma fibrinogen level	mg/dL	170–280	NA	70–130	<70
Hematocrit	%	38–44	40–55	50–60	50–65
Serum iron level	µg/dL	120–340	NA	195–380	NA

[1)] PNPA, Port of Nagoya Public Aquarium; [2)] NA, not applicable. The reference values for PNPA in bottlenose dolphins and beluga whales were established based on retrospective analysis of blood test results and clinical signs collected between 2020 and 2025.

Reference

Bossart GD, Reidarson TH, Dierauf LA, Duffield DA (2001) Clinical pathology. In: Dierauf LA, Gulland FMD (eds) CRC Handbook of Marine Mammal Medicine, 2nd edn. CRC Press: Boca Raton, pp. 383–436.

Online Resource S2. Sedation for bottlenose dolphins (*Tursiops truncatus*) and beluga whales (*Delphinapterus leucas*)

ID	Examined day (Day)	Oral sedation (mg/kg)		Intramuscular sedation (mg/kg)	
		Diazepam	Tramadol	Midazolam	Butorphanol
Bottlenose dolphins					
TT-Hp	-525	0.18	0.11	NA	NA
	0	NA	NA	0.050	0.034
	59	NA	NA	0.044	0.024
TT-LL	-735	0.39	0.22	NA	NA
	-20	0.41	NA	0.063	0.033
	0	0.41	NA	0.063	0.033
	70	0.38	NA	0.063	0.033
TT-Sr	0	0.18	0.11	NA	NA
	37	0.18	0.11	NA	NA
	72	NA	NA	0.051	0.026
Beluga whales					
DL-9	0	0.40	NA	0.104	0.050
	112	0.37	NA	0.103	0.051
DL-11	-267	NA	NA	0.093	0.059
	0	NA	NA	0.090	0.061
	56	NA	NA	0.089	0.060
DL-12	0	NA	NA	0.051	0.025
	84	NA	NA	0.052	0.026
	294	NA	NA	0.050	0.024
	433	NA	NA	0.050	0.025
	658	NA	NA	0.050	0.025

In TT-HP, TT-LL, TT-SR, and DL-11, respiratory infections were suspected based on their results of the clinical examinations as described in the text, the bronchoscopy was carried out. The first day on which respiratory infection was diagnosed by bronchoscopy in each animal was designated as Day 0, and dates prior to this were indicated with negative numbers. For animals that underwent bronchoscopy as part of routine health monitoring (DL-9, DL-12), the first bronchoscopy performed during the study period was designated as Day 0. NA, not applicable.

Online Resource S3. Clinical signs of cetaceans used in this study

ID	Sex	Birth	Age on Day 0 (years old)	Average rectal temperature (℃)	Condition on Day 0		Diagnosed status	
					Rectal temperature (℃) highest within Day 0 ±1	Appetite (%)	Diagnosed diseases	Major microorganisms for respiratory infection
Bottlenose dolphin								
TT-HP	Female	23-Sep-17	6	36.4	37.3	100.0	Respiratory bacterial infection<br>Respiratory fungal infection<br>Congenital scoliosis<br>Tail fin wounds<br>Iron deficiency anemia	*Escherichia coli*<br>*Candida albicans*
TT-LL	Female	1997 [1)]	27	36.6	37.0	100.0	Respiratory fungal infection	*Candida albicans*<br>*Aspergillus fumigatus*
TT-SR	Male	21-Aug-16	6	36.5	37.0	57.9	Respiratory bacterial infection	*Escherichia coli*
Beluga whale								
DL-9	Female	25-Jul-07	17	35.3	35.6	100.0	Clinically healthy	NA
DL-11	Male	02-Aug-12	12	34.9	34.9	100.0	Respiratory bacterial infection<br>Iron deficiency anemia	*Escherichia coli*<br>*Pseudomonas aeruginosa*
DL-12	Male	2007 [2)]	15	34.9	35.0	100.0	Clinically healthy	NA

Years of birth were estimated by body length in a bottlenose dolphin [1)] (Kasuya et al., 1997) and a beluga whale [2)] (Vos et al., 2020). In TT-HP, TT-LL, TT-SR, and DL-11, respiratory infections were suspected based on their results of the clinical examinations as described in the text, the bronchoscopy was carried out. The first day on which respiratory infection was diagnosed by bronchoscopy in each animal was designated as Day 0, and dates prior to this were indicated with negative numbers. For animals that underwent bronchoscopy as part of routine health monitoring (DL-9, DL-12), the first bronchoscopy performed during the study period was designated as Day 0. NA, not applicable.

References

Kasuya T, Izumisawa Y, Komyo Y, Ishino Y, Maejima Y (1997) Life history parameters of bottlenose dolphins off Japan. IBI Rep 7: 71–107 (In Japanese with English Summary)

Vos DJ, Shelden KEW, Friday NA, Mahoney BA (2020) Age and growth analyses for the endangered belugas in Cook Inlet, Alaska. Mar Mamm Sci 36: 293–304. http://dx.doi.org/10.1111/mms.12630

Online Resource S4. Isolated microorganisms from bronchoalveolar lavage fluid and blow samples

ID	Examined day (Day)	Bronchoalveolar lavage fluid									Blow sample								
		Bacteria						Fungi			Bacteria						Fungi		
		Species	Susceptibility					Species	Susceptibility		Species	Susceptibility					Species	Susceptibility	
			MINO	FRPM	LEVO	AMC	AMK		ITCZ	VRCZ		MINO	FRPM	LEVO	AMC	AMK		ITCZ	VRCZ
Bottlenose dolphins																			
TT-Hp	-525	Negative						Negative			*Escherichia coli*	S	S	R	R	S	Negative		
											Shewanella putrefaciens	S	R	S	R	S			
											Morganella morganii	R	I	S	R	S			
	0	*Escherichia coli*	S	I	I	S	S	*Candida albicans*	S	S	*Escherichia coli*	S	S	R	S	S	*Candida albicans*	S	S
		Proteus hauseri	R	S	S	R	S				*Proteus hauseri*	R	S	S	R	S			
											Pseudomonas aeruginosa	N	R	R	N	S			
	59	*Escherichia coli*	I	I	I	S	S	Negative			*Escherichia coli*	S	S	R	S	S	*Aspergillus* -like filamentous fungi	NA	NA
		Pseudomonas aeruginosa	N	S	R	N	S				*Proteus hauseri*	R	S	S	R	S			
											Shewanella putrefaciens	S	R	S	R	S			
TT-LL	-735	*Escherichia coli*	R	R	R	R	S	*Aspergillus* -like filamentous fungi	NA	NA	*Escherichia coli*	R	S	R	R	S	Negative		
	-20	Negative						Negative			*Enterococcus faecalis*	I	S	R	S	N	*Candida albicans*	R	R
											Vibrio alginolyticus	S	S	S	S	S			
	0	Negative						*Candida albicans*	S	S	*Enterococcus faecalis*	R	S	R	S	N	*Candida* spp.	NA	NA
								Aspergillus fumigatus	I	S	*Vibrio alginolyticus*	S	S	S	S	S			
											Vagococcus spp.	S	I	R	I	N			
	70	Negative						Negative			*Enterococcus faecalis*	R	S	R	S	N	Unidentified fungi	NA	NA
											Providencia rettgeri	R	S	S	R	S			
TT-Sr	0	*Escherichia coli*	R	S	S	S	S	Negative			*Escherichia coli*	*R*	*S*	*S*	*S*	*S*	*Candida guilliermondii*	S	S
											Vibrio alginolyticus	*S*	*S*	*S*	*S*	*S*			
	37	*Escherichia coli*	R	S	R	S	S	Negative			Negative						Negative		
	72	Negative						Negative			Negative						Negative		
Beluga whales																			
DL-9	0	Negative						Negative			*Shewanella putrefaciens*	S	R	S	R	S	*Aspergillus* -like filamentous fungi	NA	NA
											Kocuria spp.	S	S	S	S	S			
	112	Negative						Negative			*Vibrio* spp.	S	S	S	S	S	Negative		
DL-11	-267	Negative						Negative			*Proteus vulgaris*	S	S	S	S	S	Negative		
											Vagococcus spp.	I	I	R	I	N			
											Serratia liquefaciens	R	S	S	S	S			
	0	*Pseudomonas aeruginosa*	N	R	S	N	S	Negative			*Pseudomonas aeruginosa*	N	R	S	N	S	Negative		
		Escherichia coli	S	S	R	S	S				*Proteus vulgaris*	R	S	S	R	S			
		Morganella morganii	R	S	S	R	S												
		Shewanella putrefaciens	S	R	S	R	S												
		Vibrio alginolyticus	S	S	S	S	S												
	56	*Escherichia coli*	S	S	R	S	S	Negative			*Escherichia coli*	S	S	R	S	S	Negative		
											Shewanella putrefaciens	S	R	R	R	S			
											Vibrio spp.	S	S	S	S	S			
DL-12	0	Negative						Negative			Negative						Negative		
	84	Negative						Negative			Negative						Negative		
	294	Negative						Negative			Negative						*Candida* spp.	NA	NA
	433	Negative						Negative			*Corynebacterium* spp.	S	S	S	S	S	Negative		
	658	Negative						Negative			Negative						Negative		

In TT-HP, TT-LL, TT-SR, and DL-11, respiratory infections were suspected based on their results of the clinical examinations as described in the text, the bronchoscopy was carried out. The first day on which respiratory infection was diagnosed by bronchoscopy in each animal was designated as Day 0, and dates prior to this were indicated with negative numbers. For animals that underwent bronchoscopy as part of routine health monitoring (DL-9, DL-12), the first bronchoscopy performed during the study period was designated as Day 0. MINO, minocycline; FRPM, faropenem; LEVO, levofloxacin; AMC, amoxicillin-potassium clavulanate, AMK, amikacin; S, susceptible; I, intermediate; R, resistant; N, not tested

Online Resource S5. Prescriptions for bottlenose dolphins (*Tursiops truncatus*) and a beluga whale (*Delphinapterus leucus*)

ID	Medicine	Dosage	Prescription (Day)	
			Start	Stop
Bottlenose dolphins				
TT-HP	Minocycline (Sawai, Osaka, Japan)	2.1 mg/kg P.O. BID	-54	32
			59	141
	Ferrous sulfate (Mylan EPD, Tokyo, Japan)	1.5 mg/kg P.O. SID	-39	59
	Voriconazole (DSEP, Tokyo, Japan)	2.7 mg/kg P.O. BID [1)]	4	59
		3.4 mg/kg P.O. BID [2)]		
	PHMB and betaine (Prontosan, B. Braun Medical AG, Sempach, Switzerland)	20 minutes, three times a week	10	141
TT-LL	Voriconazole	3.0 mg/kg P.O. BID [1)]	10	68
		3.0 mg/kg P.O. BID [2)]		
TT-SR	Minocycline	2.0 mg/kg P.O. BID	-7	2
	Itraconazole (Kaken, Tokyo, Japan)	3.1 mg/kg P.O. BID	2	41
	Faropenem (Maruho, Osaka, Japan)	5.2 mg/kg P.O. BID	3	76
	Levofloxacin (Sawai, Osaka, Japan)	5.5 mg/kg P.O. SID	6	41
Beluga whales				
DL-11	Ferrous sulfate	1.1 mg/kg P.O. BID	-109	-68
			57	132
	Amoxicillin (Fujita, Tokyo, Japan)	10.4 mg/kg P.O. BID	-60	4
	Potassium clavulanate (GSK, Tokyo, Japan)	2.1 mg/kg P.O. BID	-18	4
	Levofloxacin	4.9 mg/kg P.O. SID	5	56
	Amikacin (FujiPharma, Toyama, Japan)	0.78 mg/kg I.H. q2d	20	40
		0.78 mg/kg I.H. SID	42	56

In TT-HP, TT-LL, TT-SR, and DL-11, respiratory infections were suspected based on their results of the clinical examinations as described in the text, the bronchoscopy was carried out. The first day on which respiratory infection was diagnosed by bronchoscopy in each animal was designated as Day 0, and dates prior to this were indicated with negative numbers. For animals that underwent bronchoscopy as part of routine health monitoring (DL-9, DL-12), the first bronchoscopy performed during the study period was designated as Day 0. The dosage was based on previously reported data for cetaceans (Simeone and Stoskopf, 2018). The medicines were prescribed [1)] three days continuously as a loading dose, and [2)] every 6–18 days as a maintenance dose with checking plasma voriconazole concentrations. P.O., per os; SID, semel in die; BID, bis in die; I.H., inhalation; q2d, quaque 2 die;PHMB, polyhexamethylene biguanide.

Reference

Simeone CA, Stoskopf MK (2018) Pharmaceuticals and formularies. In: Gulland FMD, Dierauf LA, Whitman KL (eds) CRC Handbook of Marine Mammal Medicine, 3rd edn. CRC Press, Boca Raton. pp. 607–673.
